# The Effect of Periodontal Disease on Metabolic Control in Patients With Diabetes Mellitus in South Africa: Protocol for a Systematic Review

**DOI:** 10.2196/27471

**Published:** 2021-07-22

**Authors:** Anthea Jeftha, Tina Roberts, Faheema Kimmie-Dhansay

**Affiliations:** 1 Department of Oral Medicine and Periodontology Faculty of Dentistry University of the Western Cape Cape Town South Africa; 2 Department of Oral Pathology University of the Western Cape Cape Town South Africa; 3 Research Support Unit University of the Western Cape Cape Town South Africa

**Keywords:** periodontal disease, periodontitis, bleeding on probing, type 2 diabetes mellitus, fasting glucose, HbA1C, South Africa

## Abstract

**Background:**

The increase in the prevalence of type 2 diabetes mellitus (T2DM) and its associated complications is burdensome to the South African health system. Understanding the role of comorbid diseases, such as periodontal disease (PD), and their effect on metabolic control in patients with DM in South Africa will raise awareness about the importance of periodontal interventions among patients with DM in South Africa.

**Objective:**

The review will aim to determine the effect of PD on the metabolic control of T2DM in a South African population.

**Methods:**

A systematic review of the relationship between PD and metabolic control in patients with T2DM in a South African population will be conducted. Cohort, cross-sectional, and case-controlled studies will be considered in which the outcome of interest is diabetic control. A search will be done in the following sources: EBSCOhost (academic search complete; dentistry and oral sciences), PubMed, ScienceDirect, and the South African National ETD Portal for articles published in English. There will be no limit placed on the date of the publication. The reference list of articles will be reviewed for further inclusion of critical articles. Two independent reviewers (AJ and FK-D) will do study selection, data extraction, and quality analysis. All disputes will be resolved by discussion, and the entire team will verify final decisions.

**Results:**

The systematic review protocol was registered in the International Prospective Register of Systematic Reviews (PROSPERO). A preliminary search was conducted using the keywords ((perio*) AND (diabet*)) AND (“South Africa”). The review process should be completed by December 2021.

**Conclusions:**

The review will determine the effect of PD on metabolic control in patients with T2DM in South Africa. The outcome would inform health policy to highlight the need to include periodontal care into treatment protocols in patients with T2DM. In this process, the feasibility for future research in this area of interest will also be defined.

**Trial Registration:**

PROSPERO International Prospective Register of Systematic Reviews CRD42020221064; https://www.crd.york.ac.uk/PROSPERO/display_record.php?RecordID=221064

**International Registered Report Identifier (IRRID):**

PRR1-10.2196/27471

## Introduction

### Rationale

Acute and chronic inflammation of the attachment apparatus around teeth, the periodontium, due to dysbiosis in the oral microbiome, constitutes what is known as periodontal diseases (PDs) [1]. Inflammation limited to gingivae is known as gingivitis, whereas an extension of the inflammatory infiltrate can result in clinical attachment loss or periodontitis [2]. The progression to periodontitis depends on the host’s susceptibility and the host’s response to the dental biofilm [1].

Diabetes mellitus (DM) constitutes a group of diseases hallmarked by chronic hyperglycemia and is classified by etiology [3] into type 1 DM (T1DM), type 2 DM (T2DM), other specific types, and hyperglycemia first detected in pregnancy [3]. T1DM is caused by the autoimmune destruction of β cells of the pancreas. It constitutes 5%-10% of all diagnoses of DM, but has a higher prevalence in children diagnosed with DM (80%-90%) [4]. Insulin resistance is the hallmark of T2DM, and is also responsible for concurrent metabolic conditions that may occur simultaneously with T2DM [5]. T2DM represents 90%-95% of all diagnosed cases of DM [3]. A variety of conditions of mostly genetic origin, as well as related to drug therapy, have been included under other specific types of DM. Gestational DM is a potential precursor to the development of T2DM later in life and is classified under hyperglycemia first detected in pregnancy [6]. It is characterized by the inability to tolerate glucose during pregnancy, with no prior history of glucose intolerance. Adverse pregnancy outcomes may be associated with gestational DM [5].

South Africa has an increasing population of patients with T2DM [7], resulting in an increased health care burden. In 2000, it was reported that DM in South Africa accounted for approximately 14% of cases of ischemic heart disease, 12% of hypertensive disease, 12% of renal disease, and 10% of stroke [8]. Furthermore, the complications of T2DM are associated with a poor health-related quality of life [5]. These complications and their effects result from poor metabolic control that can be prevented or minimized by well-timed intervention strategies.

Currently, DM is the fourth highest combined cause of death and disability in South Africa [9]. Data from similarly classified income countries matched for socioeconomic demographics show that DM-related death and disability is significantly higher in South Africa [9]. In 2015, T2DM was the second largest cause of death in the South African population. It was also the leading cause of death in women in the same year [9]. Underdiagnosed DM and lifestyle risk factors such as obesity, urbanization, and limited access to health care have been cited as possible causes of the high diabetes-related mortality rate in the South African context [7].

Periodontitis and DM are established comorbid diseases [10]. A recent review reported poorer periodontal outcomes in patients with uncontrolled DM [11]. The study showed that patients with diabetes having concomitant periodontitis had increased glucose impairment and insulin resistance. Furthermore, the authors reported that patients with DM were 3 times more likely to develop periodontitis than those without. The investigation also showed that the incidence of DM and diabetic complications was higher in patients with periodontitis. Sanz et al [11] reported that the treatment of periodontitis improves serum HbA_1C_ levels in patients with DM [11]. The mechanistic links to the comorbid relationship between periodontitis and DM are primarily due to inflammation [12]. In vitro and in vivo studies have shown increased levels of proinflammatory cytokines in patients with poorly controlled diabetes and in those with diabetes and concimitant periodontitis [13]. These cytokines, tumor necrosis factor-α, other inflammatory markers such as C-reactive protein, and mediators of oxidative stress burden DM control, and have been shown to decrease after a periodontal intervention [13].

The South African context is unique due to the disparity between the socioeconomic groups, which is essentially a result of decades of laws that enforced racial segregation and disparate economic development in South Africa. These circumstances have resulted in socioeconomic segregation. Currently, South Africa has the highest income disparity according to the Gini Index (63.0) [14]. This disparity has caused unequal access to quality health care, as the public health system, with limited resources, services most of the population. In addition, South Africa has limited resources to manage the costs of diabetes, as the country is already overwhelmed with a quadruple burden of disease resulting from high infectious disease rates, noncommunicable disease, and maternal and child mortality [15]. Therefore, determining the influence of comorbid diseases such as PD on DM is important in the South African society so that protocols and standards of care can be influenced across the board to limit the unneccesary burden placed on the already overextended health care system.

### Objectives

This review will determine the effect of PD on metabolic control in adults with T2DM in South Africa. Should PDs prove to have an adverse effect on metabolic control in T2DM in South Africa, this review will highlight the importance of PD intervention in this patient group. The latter will form the basis for policy development and augment holistic treatment strategies for T2DM in South Africa.

## Methods

### Overview

This systematic review will synthesize data to determine whether PDs affect metabolic control in patients with T2DM in South Africa. The proposed review will be conducted according to the requirements contained within the Preferred Reporting Items for Systematic Review and Meta-Analysis Protocols (PRISMA-P) checklist (Multimedia Appendix 1) for systematic review and meta-analysis protocol [16] and has been registered with PROSPERO (CRD42020221064).

### Eligibility Criteria

Eligible studies that report on the metabolic control in South African patients with T2DM having PD will be reviewed. Metabolic control outcomes will be measured by HbA_1C_ and fasting glucose levels. Only English publications will be included and there will be no date limitation for the eligible publications. Unpublished studies such as theses and dissertations will also be sourced. Reference lists of identified publications will be reviewed to find additional articles that may satisfy the eligibility criteria. The following study designs will be included in this review: cohort, cross-sectional, and case-controlled studies.

### Information Sources

The following electronic databases will be searched for publications and unpublished studies that meet the eligibility criteria for this review: EBSCOhost (academic search complete; dentistry and oral sciences), PubMed, ScienceDirect, and the South African National ETD Portal. Authors will be contacted should any further data be required. All articles retrieved as of March 8, 2021, will be included for this review.

### Search Strategy

An initial limited search of MEDLINE was undertaken to identify articles pertaining to the topic. The text words in the titles and abstracts and terms used to describe and index the relevant articles were used to develop a complete search strategy. An example of a search strategy outlined for PubMed can be found in Multimedia Appendix 2. The search strategy, including all identified keywords and index terms, will be adapted for each included database or information source. Studies will be limited to the English language and human patients only. The reference list of all included sources of evidence will be screened for additional studies. The results of the search will be recorded in a data capturing (Multimedia Appendix 3) sheet to include the source, the date of search, the number of hits, and a reference link to the articles.

### Study Selection, Data Management, and Data Collection Process

The eligibility criteria will be used to guide 2 reviewers (AJ and FK-D), who will select studies for inclusion. Selections will be recorded in Rayyan [17], which will be used to manage records and resolve duplications.

Study selection will be blinded, and any disagreements will be resolved by discussion. A data extraction tool (Multimedia Appendix 4) will be used to guide reviewers on the data that would need to be extracted from the included articles.

### Data Items

The extracted data will be recorded in duplicate by 2 independent reveiwers (AJ and FK-D) who will be blinded. An MS Excel spreadsheet will be used to capture extracted data that will include publication details, the setting of research study, age of the population, sex, PD clinical determinants, number of cases, total sample size, and diabetic control as measured by blood glucose or HbA_1C_ levels. PD clinical determinants will include bleeding index or bleeding on probing percentage, clinical attachment loss, and pocket depths.

### Outcomes and Prioritization

The primary outcome is to determine the metabolic control of adults with T2DM in South Africa who have PD. HbA_1C_ and fasting glucose levels will be used as the primary measure of metabolic control in T2DM.

### Risk of Bias in Individual Studies

To assess the risk of bias within included studies, the methodological quality of potential studies will be evaluated using the Joanna Briggs Institute (JBI) criteria for assessing the quality of nonrandomized studies (case-controlled, cross-sectional, or cohort) in meta-analyses. An 8-point item scale will be used to evaluate the risk of bias in a cross-sectional study using the JBI criteria. A cutoff point of 5 will indicate a low risk of bias. A 10- and 11-point item scale will be used to evaluate a case-controlled and cohort study, respectively. A minimum cutoff of 6 and 7 for case-controlled and cohot studies, respectively, will indicate a low risk of bias. Two separate reviewers (AJ and FK-D) will undertake the assessments and a third reviewer (TR) will be used as an arbiter to resolve any disagreements. Reveiwers will be blinded throughout the process.

### Data Synthesis

Studies with homogenous designs will be subjected to a meta-analysis. The choice of the model (fixed or random) and the method for meta-analysis will be based on the guidance by Tufanaru et al [18] and Moola et al [19].

Continuous outcomes will be analyzed using weighted mean differences (with 95% CI) or standardized mean differences (95% CI) if different measurement scales are used. Skewed data and nonquantitative data will be presented descriptively.

When there are missing data, an attempt will be made to contact the author(s) of the original study to obtain the relevant missing information. If missing data cannot be obtained, an imputation method will be used. Important numerical data will be evaluated with care.

### Assessment of Heterogeneity

Clinical heterogeneity will be tested by considering the variability in participant factors among trials (eg, age) and trial factors (randomization concealment, blinding of outcome assessment, losses to follow-up, treatment type, co-interventions). Statistical heterogeneity will be tested using the chi-square test (significance level: *P*=.1) and *I*^2^ statistic (0%-40%, might not be important; 30%-60%, may represent moderate heterogeneity; 50%-90%, may represent substantial heterogeneity; 75%-100%, considerable heterogeneity). There will be no *I*^2^ cutoff point to assess heterogeneity. Any source of heterogeneity will be explored using subgroup or sensitivity analysis.

A meta-analysis of studies with similar comparisons reporting the same outcomes will be conducted. The results from studies not suitable for inclusion will be reported in a table. The meta-analysis will be performed using Stata 16 (StataCorp). If appropriate, a subgroup analysis of age groups may be performed.

The outcome will be combined and calculated using the statistical software Stata 16, according to the statistical guidelines referenced in the current version of the Cochrane Handbook for Systematic Reviews of Interventions [20]. The Mantel–Haenszel method will be used for the fixed-effect model if tests of heterogeneity are not significant. If statistical heterogeneity is observed (*I*^2^≥50% or *P*<.1), the random effects model will be chosen. If heterogeneity is substantial, a meta-analysis will not be performed; a narrative, a qualitative summary will be done instead.

Effect sizes expressed as odds ratios or relative risk or other association measures and their 95% CIs will be calculated for analysis. Where effect estimates and standard errors are unavailable, they will be calculated from crude data and 95% CIs.

### Metabias

A funnel plot will be generated within Stata 16 to assess publication bias if 10 or more studies were included in a meta-analysis. Statistical tests for funnel plot asymmetry (Egger test, Begg test) will be performed where appropriate.

### Confidence in Cumulative Evidence

The quality of evidence for all outcomes will be assessed using GRADE (Grading of Recommendations Assessement, Development and Evaluation).

## Results

This review will be conducted from September 2021 to December 2021. The PROSPERO registration number is CRD42020221064. Two reviewers (AJ and FK-D) will be blinded at each stage of the process. Regular team meetings will be held to settle any disputes or differences between the inclusions of the reviewers. This will serve to enhance the transparency of the review process. All discussions will be recorded as evidence. There is no conflict of interest within the team. Once the systematic review is completed, it will be submitted for publication.

## Discussion

Although reviews from elsewhere have shown that PDs increased blood glucose levels in patients with T2DM, and that the treatment of PD has shown an improvement in metabolic control in this group of patients, a systematic review in the South African population had not yet been conducted to consolidate findings in this population [10].

A study conducted in India determined that periodontitis, measured by periodontal inflammatory surface area, was more associated with elevated blood glucose levels and retinopathy and nephropathies [21]. In that same study, patients with DM without periodontitis had significantly better metabolic control than those with chronic periodontitis [21]. India’s socioeconomic background is similar to that of South Africa, and therefore, similarities can be expected between these 2 populations.

Although the importance of PD diagnosis and management in T2DM is well-documented elsewhere [11], the implementation has not been widely accepted in South Africa. To our knowledge, this will be the first systematic review conducted to evaluate the effect of PD on metabolic control in T2DM in South Africa.

Given the comorbid relationship between the 2 diseases, and the increasing burden of T2DM on the South African population, this review’s relevance is emphasized.

The feasibility of future research on PD and DM in South Africa will be determined. Outcomes will also include the guidance of policy for the intervention and prevention of PD in patients with DM based on evidence produced by this review. Any required protocol modifications will be discussed by all reviewers and implementation of justifiable modifications will be documented and reported in the review.

## Appendix

Multimedia Appendix 1 PRISMA-P checklist.

Multimedia Appendix 2 Search strategy.

Multimedia Appendix 3 Database retrieval tool.

Multimedia Appendix 4 Data extraction tool.

## Abbreviations

DM: diabetes mellitus
GRADE: Grading of Recommendations Assessement, Development and Evaluation
JBI: Joanna Briggs Institute
PD: periodontal diseases
PRISMA-P: Preferred Reporting Items for Systematic Review and Meta-Analysis Protocols
T1DM: type 1 diabetes mellitus
T2DM: type 2 diabetes mellitus
