# Supplementary figures and images for: The Effect of Periodontal Disease on Metabolic Control in Patients With Diabetes Mellitus in South Africa: Protocol for a Systematic Review
Source: JMIR Res Protoc. 2021 Jul 22;10(7):e27471. doi: 10.2196/27471 (PMC8367174; doi:10.2196/27471)

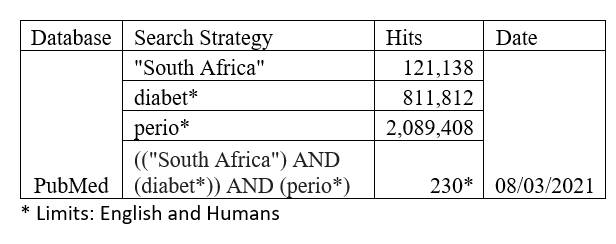

Supplement: Multimedia Appendix 2 [file resprot_v10i7e27471_app2.png]

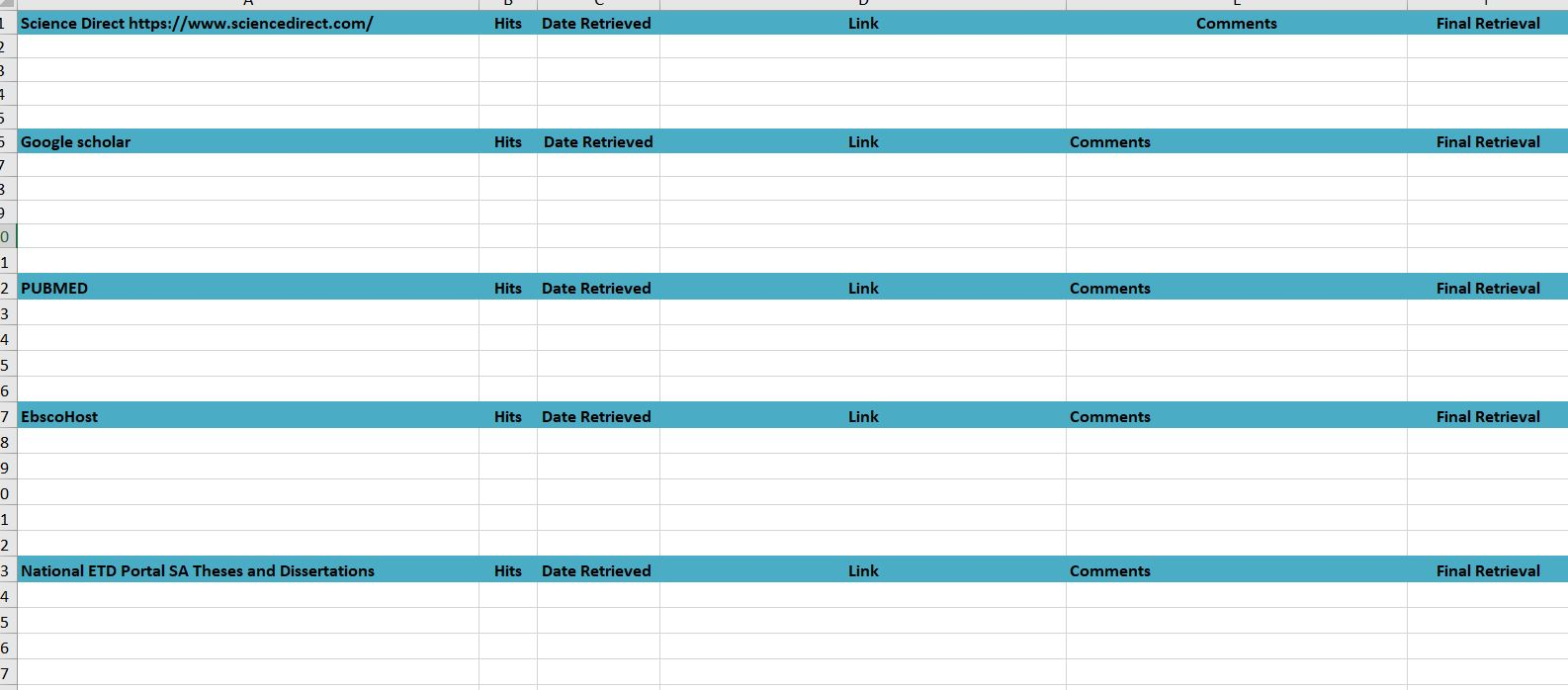

Supplement: Multimedia Appendix 3 [file resprot_v10i7e27471_app3.png]

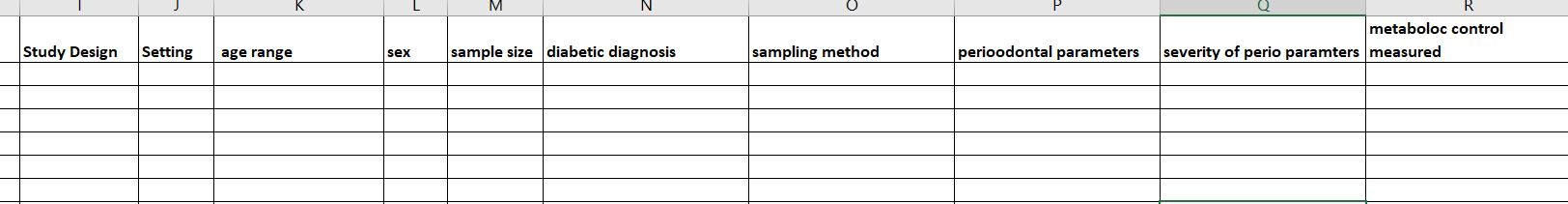

Supplement: Multimedia Appendix 4 [file resprot_v10i7e27471_app4.png]
